# Label-Free Enrichment of Functional Cardiomyocytes Using Microfluidic Deterministic Lateral Flow Displacement

**DOI:** 10.1371/journal.pone.0037619

**Published:** 2012-05-29

**Authors:** Boyang Zhang, James V. Green, Shashi K. Murthy, Milica Radisic

**Affiliations:** 1 Department of Chemical Engineering and Applied Chemistry, University of Toronto, Toronto, Ontario, Canada; 2 Department of Chemical Engineering, Northeastern University, Boston, Massachusetts. United States of America; 3 Institute of Biomaterials and Biomedical Engineering, University of Toronto, Toronto, Ontario, Canada; Foundation for Applied Medical Research, Spain

## Abstract

Progress in cardiac cell replacement therapies and tissue engineering critically depends on our ability to isolate functional cardiomyocytes (CMs) from heterogeneous cell mixtures. Label-free enrichment of cardiomyocytes is desirable for future clinical application of cell based products. Taking advantage of the physical properties of CMs, a microfluidic system was designed to separate CMs from neonatal rat heart tissue digest based on size using the principles of deterministic lateral displacement (DLD). For the first time, we demonstrate enrichment of functional CMs up to 91±2.4% directly from the digested heart tissue without any pre-treatment or labeling. Enriched cardiomyocytes remained viable after sorting and formed contractile cardiac patches in 3-dimensional culture. The broad significance of this work lies in demonstrating functional cell enrichment from the primary tissue digest leading directly to the creation of the engineered tissue.

## Introduction

There are nearly 16 million people in North America alone suffering from coronary heart disease (CHD) with additional 770,000 new cases occurring annually contributing to $156 billion in medical expenses and lost productivity per year [1]. The majority of the cases involve at least one myocardial infarction (MI) event. During MI, a regional blockage in the coronary arteries constricts blood perfusion downstream which causes death of cardiomyocytes (CMs) in the infarct zone. Depending on the severity of infarction up to one billion CMs could be lost in the infarct zone [2]. These cells cannot be readily replaced by the heart since the adult mammalian CMs are considered to be terminally differentiated, have extremely low proliferation rates [3] and low turn-over rates in humans [4]. Instead, a non-contractile scar forms that consists of fibroblasts and extracellular matrix. Ultimately, a pathological remodeling process of the heart leads to the thinning of the ventricular wall, dilatation of the ventricle and diminishing ability of the heart to pump blood. Cell replacement therapies offer the possibilities to develop new therapies for MI by replacing CMs lost during an MI either through cell injection or implantation of engineered cardiac patches.

Although non-myocyte cell types such as bone marrow cells have been tested in clinical trials and demonstrated to exert beneficial effects by improving vascularization and acting on the myocardium through paracrine mechanisms and secretion of growth factors [2], [5]–[10] the replacement of contractile cells, CMs is required for true regeneration. The first evidence that cell injection may be a viable therapeutic approach for MI came from rodent studies with injection of fetal or neonatal CMs. CM injection improved left ventricular function and ventricle thickness, thus attenuating pathological remodeling following MI [11]–[14]. Injected CMs were demonstrated to integrate through gap junctions and intercalated discs with the host CMs [15]. Our group has worked extensively on growing patches of functional cardiac tissue *in vitro* [16]. Cardiac tissue patches consisting of scaffolds or hydrogels and primary rat CMs have been shown to mediate cardiac function following myocardial infarction in rats [17]–[18]. Recent advances in stem cell biology offer an unprecedented opportunity to generate millions of human CMs from either embryonic stem cells (ESCs) [19] or induced pluripotent stem cells (iPSCs) [20] to be used for cardiac cell therapy.

However, efficient cell separation methods are lacking. One of the major challenges in growing functional tissues with defined cell composition is the availability of purified cells [21]. It is generally accepted that tri-culture of CMs, endothelial cells and fibroblasts enhances engineered cardiac tissues in vitro [22], [23] and enables their survival in vivo [24], [25]. However, providing defined tri-culture requires first obtaining a homogenous cell population from a heterogeneous cell source. Since all of the known CMs markers are intracellular proteins, antibody staining (e.g. for cardiac troponin I, [26]) or genetic labeling (e.g. neomycin resistance under control of a myosin heavy chain promoter [27]) have so far been used for identification of CMs and their separation. Antibody staining of intracellular markers such as contractile proteins requires cell permeabilization which unfortunately renders the cells non-viable and unusable for cardiac therapy. On the other hand, genetic labeling of cells for clinical applications cannot be performed in humans due to ethical concerns.

Besides genetic manipulation, other characteristics of CMs have also been explored as a basis for separation. Compared to other cell types, CMs tend to contain more mitochondria in order to sustain the energy requirement for contraction. Utilizing this difference, Hattori et al. recently enriched CMs up to 99% purity by labeling the mitochondria with a fluorescent marker, tetramethylrhodamine methyl ester perchlorate [28]. In addition, they have shown that teratoma formation was prevented when transplanting purified CMs into testes [28]. Furthermore, they have also demonstrated that this method can be applied to cells from various species including neonatal rats, mouse, and human [28]. However, the long term effect of mitochondria labeling has not been studied and cells whose intracellular components are labeled with fluorescent probes are cannot be used in clinical applications due to the unknown long-term effects of these organic probe molecules in humans.

A new report identified SRP1a as a surface marker of CMs derived from human pluripotent stem cells [29], but the wide applicability of the SRP1a antibody across the species and the high yield of CMs upon SRP1a labeling is yet to be determined. Furthermore, use of mouse-raised antibodies for cell separation in clinical applications can induce sensitization in patients and generation of anti-mouse IgGs [30]. Therefore, bearing in mind the complex health implication in future clinical studies, there is a strong incentive for label-fee cell separation with minimal cell treatment.

The most widely used label-free enrichment technique for CMs derived from primary sources such as rat hearts is pre-plating [31]. This method makes use of the different cell attachment rate between fibroblasts and CMs, where fibroblasts attach faster to the tissue culture surface compared to the CMs. This non-specific technique requires sequential plating steps each at ∼1 hr/step that are both time consuming and often lack consistency. For instance, two rounds of pre-plating are required to enrich CMs to only 80% [31]; further enrichment is not possible without significantly undermining cell viability. Thus, presently, the high-purity, label-free, separation of living CMs is virtually impossible. Therefore, alternative properties of CMs must be explored.

It is well known that CMs, which specialize to generate contractile force, occupy 80–90% of the heart volume due to their large size compared to the other cells found in the heart [32]. Utilizing microfluidic technology, a diffusive filtering method including one center channel and two side channels separated by micro-sieves to block any large cells was introduced [33]. This method was focused on the isolation of smaller non-myocytes and cardiomyocytes were not be effectively enriched. In addition, this device was prone to clogging by the larger cells and could not be operated for longer periods of time. In 2004, Huang et al. demonstrated a microfluidic post array system capable of separating submicron particles in a continuous fashion without clogging [34]. In laminar flow, such displacement can be predicted and controlled which makes this method exceptionally sensitive and consistent. This concept, termed deterministic lateral displacement (DLD), was recently used for the separation of white blood cells from red blood cells [35]. In addition, we previously demonstrated this method for the separation of large mammalian cells, such as H1975 epithelial cells from 3T3 fibroblasts [36]. In this work, for the first time we custom-designed a separation array based on the principle of DLD for the isolation of functional CMs directly from primary cardiac tissue isolated from neonatal rats. The sorted cells remained viable and were used for tissue engineering of cardiac patches.

## Results

### Design of the sorting device

**Figure 1:**
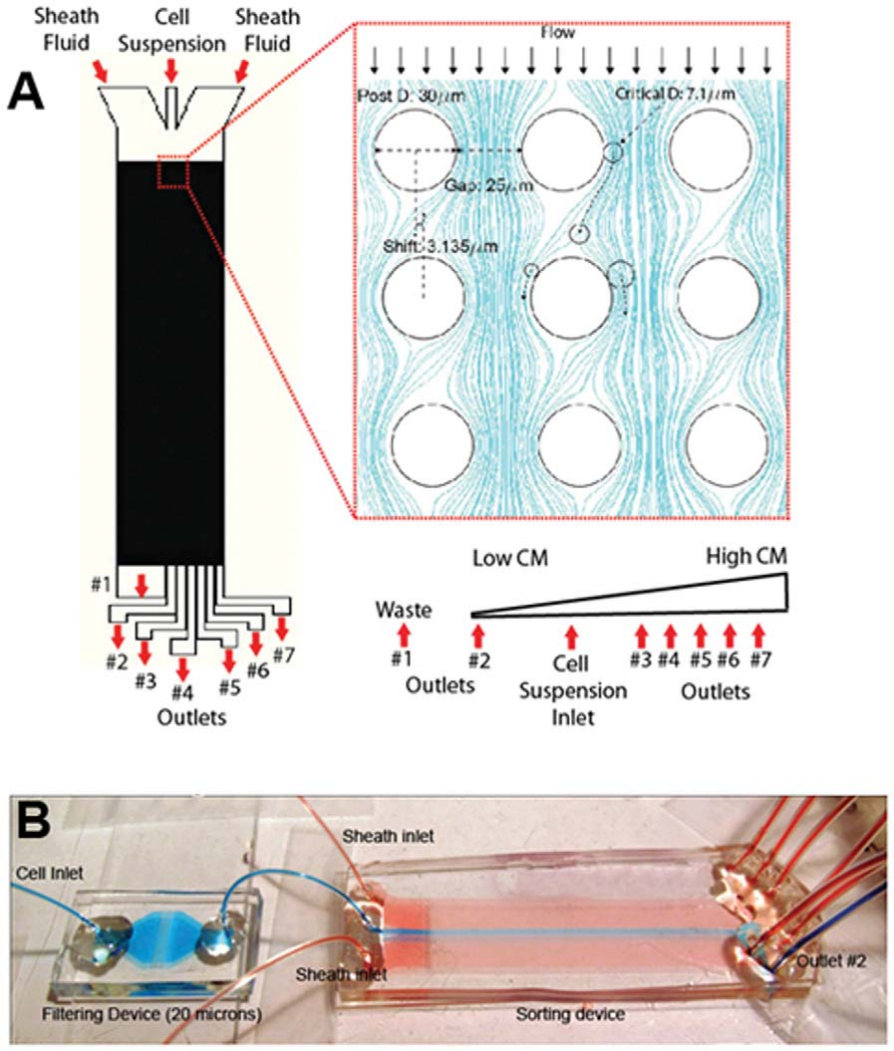
Device schematic and system setup. (A) The microfluidic sorting device is shown with the location of the inlets and outlets. Inset represents a zoom-in section of the post array with FLUENT simulation of the fluid streamlines. An illustration of expected CMs purity from each outlet is shown at the bottom. (B) An image of the system setup with color dyes illustrating stable hydrodynamic focusing. The blue dye is focused by the red dyes into the center of the chamber and exits only from outlet #2. A 20 mm filter unit is placed upstream from the sorting chamber to eliminate cell clumps.

The layout of the device is shown in **Figure 1(A)**. The microfluidic device includes two sheath fluid inlets where biological buffer, 3% (w/v) bovine serum albumin (BSA) in PBS (phosphate buffered saline), was used to focus the cells into a narrow stream. The cells focused by the sheath fluid flow through the sorting chamber (5 cm in length and 1.3 cm in width) and are collected in outlets 2–7 where outlet 2 is located in the middle of the sorting chamber. As cells move through the sorting chamber, large cells are expected to be displaced to the right side and collected in Outlets 3–7 while smaller cells are expected to stay in the middle of the chamber and collected in Outlet 2. The sorting chamber is composed of an array of posts with diameter of 30 µm. Each row of posts is slightly offset laterally from the previous row. The extent of this offset determines the critical separation diameter. Based on the cell size measurements discussed below, we determined the critical separation diameter to be 7mm. The exact device parameters to yield a critical separation diameter of 7.0 µm were determined based on the experimental correlation published by Inglis [37]. The specific post arrangement parameters are shown in Table 1.

**Table 1:** Design Parameters of the Post Array.

Critical Separation Diameter (Dc, µm)	Shift Factor (ε)	Post Gap (g, µm)	Parabolic Flow Profile Constant (η)
7.0	0.057	25	2.50

The critical separation diameter (Dc) depends on the offset of the posts and the gap size between the posts. In addition, the parabolic flow profile between adjacent posts also affects the separation diameter. The governing equation is shown as eqn (1).
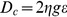
(1)


The shift factor is determined by the extent of offset by each row of posts over the center-to-center distance between two adjacent posts. The post gap is the distance between the edges of two adjacent posts. Finally, the parabolic flow profile constant is a correction factor that takes into account the parabolic velocity profile between two adjacent posts. To further illustrate the principle of the deterministic lateral displacement a FLUENT hydrodynamic simulation of the fluid streamlines around the post array was generated to demonstrate the splitting of the fluid streams by the placement of the posts (**Figure 1(A)**). Intuitively, as the offset (ε) between each row of posts increases, the amount of fluid getting diverted to the opposite side of the posts will be larger. Therefore, the fluid streams will be able to carry and divert even larger cells. This leads to an increase in critical separation diameter as predicted in eqn (1). The gap size was chosen so that the largest cells in the cell mixture can go through the post array without clogging. This theory was explained with more details in previous works [36].

Multiple external components were used to ensure proper operation of the sorting device. First, to prevent cell settling inside the syringe, a steel ball coated with PDMS and BSA was placed inside the syringe and controlled by an external magnet to provide agitation [38]. Without such agitation, cells would inevitably settle inside the syringe resulting in decreased cell output over time. Furthermore, due to their larger size and higher cell density, CMs tend to settle faster in the syringe compared to non-myocytes resulting in the reduced CMs purity from the syringe output in the absence of agitation (**Figure S1**). Second, to prevent cells from clumping inside the sorting chamber, a microfluidic filter composed of a short array of posts with 20 µm gap was placed upstream from the sorting device to filter out any cell clumps from the tissue digestion allowing only single cells to move through the system (**Figure 1B**). Lastly, a syringe pump was used to drive the flow. The syringe pump was tilted at 45 degree with the syringes pointing downward to prevent bubbles from the syringe entering the device overtime.

To test the stability of the flow driven by the syringe pump at the operating flow rate, red and blue color dyes were used to label the cell solution and the sheath solution respectively. The flow rate from the sheath inlet (500 µL/min) is nearly 6 times larger than that from the cell inlet (80 µL/min), so that the cells can be focused into a narrow stream that will only be collected by outlet 2. Stable focusing is critical in ensuring high separation purity by avoiding cross contamination. **Figure 1(B)** demonstrates the stable hydrodynamic focusing at the center of the sorting chamber. As expected red dye exited from the Outlet 2 only, demonstrating that the focusing was stable throughout the sorting chamber. The overall flow rate was chosen so that the Reynolds (Re) number at the operating condition is below 1 (Re, 0.77), which is well within the laminar flow regime where the principle of deterministic lateral displacement can be applied.

### Size distribution of primary cardiac cell mixture

**Figure 2:**
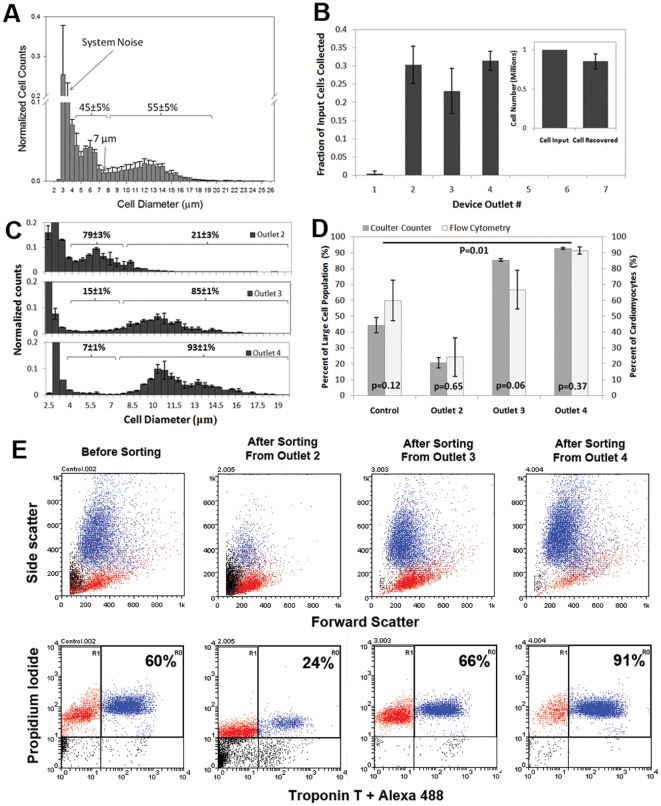
Cell separation analysis. (A) Size distribution of primary cardiac cell mixture from digested heart tissue of neonatal rat (1–2 days old) determined with Coulter Counter. Cell counts were normalized to the total number of cells counted. n = 3 (B) Fraction of total cell input collected by each outlet of the device. n = 3. (B, inset) Comparison between the total number of cells collected and the total number of input cells. (C) Cell size distribution from each outlet of the device. Cell counts were normalized to the total number of cells counted. n = 3 (D) CMs purity before and after sorting analysed by Flow Cytometry. There is a significant difference between sorted cell population from Outlet 4 and cell population before sorting, p = 0.01. n = 3 (E) Representative flow cytometry plots. Blue dots represent Troponin T (+) cells. Red dots represent Troponin T (−) cells. PI staining is used to identify nuclei. n = 3.

To enrich CMs from digested heart tissue of neonatal rats based on their size, the hydrodynamic diameter distribution of isolated primary cardiac cells in suspension was first measured. Simple techniques such as measuring the size of suspended cells with image analysis by outlining the perimeter of a cell from an image may be inaccurate and lacks consideration of the volumetric shape of the cells in 3-D. Here, a Coulter Counter was used to determine the volume of the cell by measuring the electrical impedance created by the cell passing through an aperture. The hydrodynamic diameter of the cells was subsequently derived from the volumetric measurement. The hydrodynamic diameter distribution of primary cardiac cell mixture in suspension is shown in **Figure 2(A)**. It is well known that this heterogeneous cell mixture includes two major cell populations (fibroblasts 49% and CMs 47%) and other smaller populations (smooth muscle cells 3%, endothelial cells 2%, and red blood cells) [39]. As **Figure 2(A)** shows, there are indeed two major peaks: one around 5–6 µm and another around 11–14 µm. The strong signals below 3 µm are mostly due to system noise at the lower detection limit. The area underneath the two major peaks was integrated to show that the proportion of each peak (4–7 µm, 45±5%, 7–20 µm, 55±5%) which corresponded with the reported proportion of CMs and non-myocytes [39]. The percentages were further confirmed by flow cytometry for cardiac Troponin T as described below. Since there is no method available to isolate each type of cell without permeating the cells, we were unable to determine the exact diameter of each individual live cell type. The functionality of Coulter Counter relies on non-permeated cell membranes given that cell permeation would allow electrical current to pass through the cell body and result in false measurement.

However, we hypothesized that the two peaks identified in **Figure 2(A)** represent the two major cell populations, CMs and non-myocytes (mostly fibroblasts) where the larger CMs were located around the 11–14 µm peak while the smaller fibroblasts were located around the 5–6 µm peak. Based on this hypothesis the critical separation diameter was set at 7 µm. To confirm this hypothesis, the cell mixture was first sorted based on the critical separation diameter and subsequently identified with known intracellular cardiac markers.

### Cell population analysis

After examining the fraction of total cell input collected in each outlet, it was clear that almost all the cells exited in Outlets 2–4 (**Figure 2(B**)). This indicates that the cells were displaced as far as to Outlet 4 which corresponds to approximately 3 mm in lateral displacement. Furthermore, since there were very few cells collected from Outlet 1, this indicates that there was negligible cell leakage into Outlet 1 which was designed to only collect the sheath fluid. Additionally, the total number of cells collected in all outlets of the device corresponded well to the total cell input (1 Million cells), indicating that the cell loss in the device was not significant (**Figure 2(B**, **inset**)). Thus, our further analysis focused on Outlets 2–4.

To confirm that separation was indeed based on size, the size distribution of the cells collected from Outlets 2–4 was analyzed with a Coulter Counter as shown in **Figure 2(C)**. The fractions of large cells (7–20 µm range) and small cells (4–7 µm) over the total cell number from each Outlet were determined by integrating the size distribution curve. Here, clear evidence of size dependent separation between Outlets 2 and 3 was observed and the separation diameter was ∼7 µm as expected. Furthermore, the size distribution appears to shift further to the larger end in Outlet 4 compared to Outlet 3. This demonstrates that the larger cells tend to get displaced even further laterally, as expected. In Outlet 4, 93±1% of the cells were large cells (7–20 µm) which indicates significant enrichment of large cells compared to the control with only 55±5% large cells.

To analyze the CM purity in the outputs, an intracellular marker, Troponin T, was used to identify the CMs by flow cytometry after microfluidic separation. Propidium Iodide (PI) staining was used to distinguish red blood cells from other non-myocytes. As **Figure 2(D, E)** shows, before sorting the heterogeneous cell population consisted of both Troponin T positive CMs (60±12%) and Troponin T negative non-myocytes (40±12%). After sorting, there was a clear trend of CMs enrichment from Outlet 2 to Outlet 4, with Outlet 4 producing the most enriched CMs population of 91±2%. This value was significantly higher compared to the control (cell mixture before sorting) with p = 0.01. The highest purity reached in the experiments was 94%. The flow cytometry data were also compared with the Coulter Counter data to show that the fraction of cardiomyocytes determined from the flow cytometry data closely resemble the fraction of large cells (7–20 µm) determined from the Coulter Counter data (**Figure 2D**). The yield of this separation process is 55% of the initial cardiomyocytes input. This yield was determined by taking the ratio of the total number of cardiomyocytes collected in outlet 4 to the total cardiomyocytes input.

### Functionality of enriched CMs

**Figure 3:**
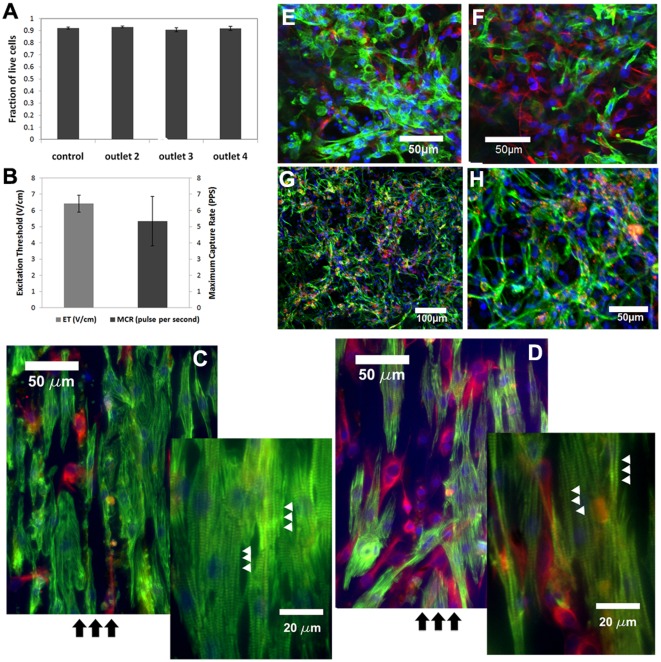
Viability and function of sorted cardiomyocytes. (A) Cell viability was measured by staining with trypan blue to confirm cell membrane integrity after sorting. The number of trypan blue negative cells was counted against the total number of cells to get the fraction of live cells. The control represents cell viability before sorting. (B) Excitation threshold and maximum capture rate measurement for cardiac patches engineered from enriched CMs. (C) Sorted CMs and (D) CMs collected after pre-plating were cultured on abraded surfaces to demonstrate cell alignment. Fluorescence image of Troponin T staining shows cell alignment and elongation in the direction of the grooves. Vimentin Staining labels the nonmyocyte population (DAPI(blue), Troponin T(green), and Vimentin (red)). Cross striations indicated by arrow heads can also be observed. Black arrow indicate the direction of the grooves. (E) Sorted CMs and (F) CMs collected after pre-plating were cultured on 3-D scaffold. Confocal images shows the CMs population (Troponin T, green) and non-myocyte population (vimentin, red)(E, F) Confocal images of sorted CMs in 3-D scaffold to show cell junction. (DAPI(blue), Troponin T(green), Connexin 43(red)).

The effects of the sorting process on the viability and functionality of the sorted cells were then characterized. First, the cells collected from each outlet were stained with trypan blue to characterize cell membrane integrity after sorting (**Figure 3(A)**). There were no significant differences in viability between the sorted cells and the control which were the unsorted cells (**Figure 3A**). Furthermore there were no significant differences between cells collected from each outlet (**Figure 3**(**A**)). Therefore, cell viability and membrane integrity were not undermined by the sorting process. Additional cell functionality tests were also performed on enriched cardiomyocytes through 2D and 3D culture.

The sorted cells were cultured on abraded surface to demonstrate cell functionality in 2-D culture [40]. As shown in **Figure 3(C, D)**, sorted cells were able to exhibit normal phenotypic responses such as elongation and cell alignment in the direction of the abraded grooves. In addition, cross-striations were visible in CMs upon Troponin T staining (**Figure 3**(**C** **, inset**)). The sorted cells were also cultured in 3-D on scaffolds to demonstrate their functionality in 3D and to characterize their synchronized contraction after 3 days of cultivation; the sorted cells connected and formed a synchronously beating cardiac patch. The excitation threshold (ET) of the cardiac patch was 6.4±0.5 V/cm with a maximum capture rate of 5.3±1.5 pulses per second consistent with previous work [41] (**Figure 3(B)**). Engineered 2D and 3D tissue from enriched CMs were also compared to those from pre-plate enriched cardiac cell mixture (Figure 3 (C–F)). Qualitatively there was significant enrichment in Troponin T positive CMs in the sorted CMs group compared to the pre-plated control. However, as expected there were still few non-myocytes mixed in with the CMs even in the sorted CMs group. This could be contributed to the initial impurity from the sorting process as well as further proliferation of non-myocytes during culture period. CMs are a terminally differentiated cell type and lack the ability to proliferate, therefore, overtime non-myocyte proliferation will gradually undermine the initially high purity of CMs in the tissue composition. Lastly, immunostaining for cardiac Troponin T and connexin-43 indicated that the patches contained elongated CMs (**Figure 3(E)**, green) connected by punctuated gap junctions (**Figure 3(F)**, red).

## Discussion

This work demonstrates the first enrichment of primary CMs directly from a primary cell source, specifically heart tissue of neonatal rats, to high purity levels without any labeling or pre-treatment of the cells. In addition, a functional cardiac patch was engineered to demonstrate functionality and contractile properties of the enriched CMs after sorting and cultivation. This is a critical step in engineering the next generation of complex cardiac tissues with precise control over cell composition. The yield of current device was determined to be around 55%. This low was caused mostly by the lost of smaller cardiomyocytes in outlet 3. The yield could potentially be improved with multiple runs by connecting multiple sorting devices in series. However, the high throughput of this system makes it possible to collect large amount of cells even at lower yield as long as the input cell number is not limited. The advantage of this microfluidic system is its ability to be scaled up with parallel processing. Therefore, the total throughput and processing time of this system is only limited by the extent of multiplexing.

Currently, with this method a single device can consistently enrich one million cells up to 91±2% within 20 min. This throughput is sufficient for cell culture studies, or for isolating cardiomyocytes from cardiac biopsies for diagnostic applications. Since this system requires only an external pump, it can be scaled-up easily to 100 devices to process up to 100 million cells within 20 min to meet large demand without significantly increasing the overall cost. Therefore this method is superior compared to conventional enrichment methods. Conventional label-free enrichment methods like pre-plating depend on sequential plating to improve purity. For instance, it takes up to two rounds of pre-plating to enrich CMs up to 80% [31]. Each round of pre-plating can last up to 75 min. Therefore, the entire process can take more than two hours. During this time viability of the CMs (the non-adherent cells) will be undermined. In fact, pre-plating is rarely performed more than two times sequentially due to the long incubation time that leads to a severe decline in viability. Therefore, CM purity up to 90% cannot be achieved via pre-plating.

Furthermore, our system requires no pre-treatment or pre-labeling to the cells which makes it ideal for clinical applications or potential integration with other microfluidic units. The described microfluidic device can be made portable and implemented in the operating room without the need for extensive cell manipulation or transport to off-site laboratories for diagnostics. Currently at the research stage, the sorting devices require few hours to fabricate and the flow system requires less than one hour to setup. If commercialized, the flow system and the sorting device can be integrated into a single system and would require minimal experience to operate and few minutes to setup. The sorting device can be reusable if it is made of glass material. The device may also be useful in label-free separation of skeletal myoblasts from primary tissues. Using engineering principles to achieve high throughput label-free separation of CMs will have a significant impact even when the CMs surface markers are fully established as expensive antibodies and the auxiliary equipment such as FACS will not be required. The broad implication of this work is the demonstration of high purity separation of functional cardiomyocytes from digested tissue through label-free method leading directly to engineered tissue.

## Methods

### Cardiac cell size distribution measurement

The hydrodynamic diameters of the cells were measured with a Beckman Z2 Coulter Counter. The 100 µm aperture was used to resolve particle size from 2 to 40 µm. The Coulter Counter scanned each cell as it passed through the aperture and recorded the electrical impedance created by the cells. This electrical signal was then converted into actual hydrodynamic diameter and plotted as a histogram. Prior to the experimental measurement, the machine was calibrated with 15 µm beads.

### Microfluidic device fabrication

Microfluidic device masters were fabricated via standard soft lithography techniques as described previously [42]. A silicon wafer was coated with SU-8 photoresist with a spin coater. The mask was drawn using AutoCAD software and printed with high resolution onto a transparency. With the mask in place, the SU-8 photoresist was exposed to 365 nm, 11 mW/cm^2^ UV light using a mask aligner (Q2001, Quintel Co., San Jose, CA). The unexposed photoresist was removed with SU-8 developer. Silicone elastomer [poly(dimethylsiloxane), PDMS] and curing agent (10∶1 ratio) were molded with the SU-8 masters at 75°C for 3 hr. Inlet and outlet holes were punched with 22-gauge needles. The replicas were plasma treated and bonded to a glass slide. Tygon tubing was press fitted into the holes.

### Experimental system setup

To prevent unspecific cell attachment during the experiment, the device was incubated with 3% BSA in PBS at room temperature for one hour prior to experiment. To prevent intercellular binding, the primary cells were also suspended in PBS with 3% BSA at a concentration of 0.33 million cells/mL. 3% BSA in PBS was also used as the sheath fluid to match the viscosity of the cells suspension. The flow rates of the sheath fluid and the cell suspension were 500 µL/min and 80 mL/min respectively. With this operating condition, the cell suspension stream was focused into a narrow stream. The flow of both sheath fluid and cell suspension was driven by an external syringe pump (Harvard Apparatus). To prevent bubbles from entering the device, the syringe pump was titled at 45 degree angle with the syringes pointing downward. The experiment took 20 to 30 min. To prevent cell settling inside the syringe during this period, a steel ball coated with PDMS and BSA was incorporated inside the syringe to generate agitation with an external magnet [38]. The solution was agitated every 2 min for 5 s. To prevent cell clumps from entering the sorting device, an on-chip filter was used. This on-chip filter was composed of three rows of posts with diameter of 30 µm, height of 40 µm and gap size of 20 µm which is larger than all the single cells but smaller than cell clumps, thus only allowing suspended single cells to go through.

### Fluid dynamic simulation

Fluid dynamic simulation of the fluid velocity profile was generated with FLUENT 6.3.26. Briefly, a repeating section of the post array was created in Gambit 2.4.6 in 3-D based on the device geometric parameters. Then, the inlet and outlet boundary conditions were estimated based on the experimental flow rates. Other boundaries were set as no-slip walls. A mesh was then generated with finer meshes near the walls and more coarse mesh away from the wall. The file was then transferred to FLUENT for simulation. The fluid density and viscosity were estimated based on the properties of water at 25°C. The simulation was solved with the built-in algorithm for continuous incompressible fluids. The mesh was refined to include approximately 2 million tetrahedral cells and convergence was reached at 10^−7^. The fluid streamlines were then plotted against the device geometrical outline.

### Heart cell isolation from neonatal rats

Neonatal rat heart tissue was digested as described using a standard isolation protocol [43]. Briefly, neonatal (1–2 day old) Sprague-Dawley rats were first euthanized. The hearts were removed and quartered. Quartered hearts were digested in 0.06% (w/v) solution of trypsin (Sigma, Canada) in Ca^2+^ and Mg^2+^ free Hank's balanced salt solution (HBSS) (Gibco, Canada) overnight at 4°C. Then, collagenase II (Worthington, USA 220 units/mL) in HBSS was used to further digest the heart at 37°C in series of five 4–8 min digestions. Right after the collagenase digestion and without pre-plating, the cells were filtered with 40 µm cell strainers and then re-suspended in 3% BSA in PBS ready for experiments. As a control cell population in 2D and 3D cell culture, the native heart cell isolate was pre-plated for 1 hr in T75 flasks. The non-adherent cells, enriched for CMs, were collected and cultivated as described below.

### Cardiomyocyte culture medium

The CMs were cultured in Dulbecco's Modified Eagle Medium (DMEM, Gibco, Canada) containing 4.5 g/L glucose, with 10% (v/v) fetal bovine serum (FBS, Gibco, Canada), 1% HEPES (100 units/mL, Gibco Canada) and penicillin-streptomycin (100 mg/mL, Gibco, Canada).

### Cell population analysis

Cell concentrations were measured with a hemacytometer. Cell collected from each outlet were first resuspended in PBS. Then the cell concentrations were measured with a hemacytometer. The total number of cell collected by each outlet was then determined by multiplying the cell concentration with the suspension volume (1 mL). The fraction of total input cells collected by each outlet were then determined and compared with the total input cell number (1 million cells) to examine the cell number loss within the sorting device. To determine the percentage of CMs before and after sorting, flow cytometry was used. For flow cytometry analysis, the cells were fixed in 4% (w/v) solution of paraformaldehyde in PBS for 15 min at room temperature and then permeated on ice with cold methanol for 2 min.

Next, the cells were labeled with Troponin T (Mouse, clone 13–11, Fisher Scientific) antibody for CM identification at dilution ratio of 1∶250 for 30 min on ice. Alexa 488 conjugated anti-mouse IgG (Sigma), was then applied at dilution ratio of 1∶200 for 30 min on ice. Propidium Iodide (VWR) staining was performed at concentration of 75 µg/mL. Stained cell suspensions were then transported on ice to Princess Margaret Hospital for flow cytometry analysis using BD FACS Calibur Flow Cytometer.

Immunostaining was performed to assess the phenotype of cultured cells. The cells were first fixed in 4% (w/v) paraformaldehyde in PBS for 15 min at room temperature. Then, the cells were permeated and blocked in 5% FBS and 0.25% Triton ×100 in PBS for 1 hour. Next, the sample was incubated in primary antibody Troponin T (Mouse, clone 13–11, 1∶200 dilution, Fisher Scientific), anti-vimentin (Mouse, 1∶200 dilution, sigma), and/or Connexin 43 (Rabbit, 1∶200 dilution, sigma) overnight at 4°C. Followed by three washes, the samples were then incubated with secondary antibodies, Alexa 488 conjugated anti-mouse IgG (1∶200 dilutions, Sigma) and/or TRITC conjugated anti-rabbit IgG (1∶200 dilution, Sigma) for 1 hour. The sample was then imaged with a fluorescence microscope (Olympus IX2-UCB, Canada) or a confocal microscope (Olympus FV5-PSU confocal with IX70 microscope, Canada).

### 2D cell culture

To determine if the cells retain functionality following the sorting, the sorted cells were seeded on polyvinyl surfaces with isotropic abraded grooves [43], [44]. Pre-plated cells served as controls. The fabrication procedure of the abraded surface was reported previously [44]. Briefly, the grooved surface was created by repeatedly abrading a plastic slide uni-directionally with 400 grit super fine sand paper (Norton Premium). The abraded surface was then cleaned with air gun and sonicated to eliminate residue debris. Prior to cell seeding, the surface was sterilized with ethanol and UV exposed overnight followed by coating with 0.2% Gelatin (Type A from porcine skin, Sigma).

### 3D cell culture

To assess the ability of the sorted cells to form functional 3-dimensional tissues, the sorted cells were seeded onto porous collagen scaffold (3 mm diameter ×300 µm thick, Ultrafoam) at a density of 10^8^cells/cm^3^ and cultured in cardiomyocyte medium. Pre-plated cells served as controls. The culture medium was replaced daily. On day 3, contractile properties were measured by field stimulation in an electrical stimulation chamber consisting of two parallel carbon electrodes spaced 1 cm apart as described [45]. Stimulation was provided by an external electric stimulator (Grass s88x). Using monophasic pulses of 2 ms duration and frequency of 1 pulse per second, the excitation threshold (minimum voltage at which synchronous contractions of 75% of the tissue in the field of view can be observed) was first determined. Then the maximum capture rate (maximum beating frequency) was determined at 200% of the determined excitation threshold voltage.

### Statistical analysis

Error bars in figures represent standard deviation. Statistical significance was determined using one-way ANOVA in conjunction with Tukey's test. Normality and equality of variance were tested. p<0.05 were considered significant. A minimum of 3 samples were used per data point.

## Supporting Information

Figure S1 **Analysis of cell output from syringes with and without magnetic stirring.**
(DOCX)

## References

[1] Rosamond W, Flegal K, Furie K, Go A, Writing Group Members (2008). Heart Disease and Stroke Statistics–2008 Update: A Report From the American Heart Association Statistics Committee and Stroke Statistics Subcommittee.. Circulation.

[2] Laflamme MA, Murry CE (2005). Regenerating the heart.. Nat Biotechnol.

[3] Soonpaa MH, Field LJ (1998). Survey of studies examining mammalian cardiomyocyte DNA synthesis.. Circulation Research.

[4] Bergmann O, Bhardwaj RD, Bernard S, Zdunek S, Barnabe-Heider F (2009). Evidence for cardiomyocyte renewal in humans.. Science.

[5] Murry CE, Field LJ, Menasche P (2005). Cell-based cardiac repair: reflections at the 10-year point.. Circulation.

[6] Orlic D, Kajstura J, Chimenti S, Jakoniuk I, Anderson SM (2001). Bone marrow cells regenerate infarcted myocardium.. Nature.

[7] Murry CE, Soonpaa MH, Reinecke H, Nakajima H, Nakajima HO (2004). Haematopoietic stem cells do not transdifferentiate into cardiac myocytes in myocardial infarcts.. Nature.

[8] Balsam LB, Wagers AJ, Christensen JL, Kofidis T, Weissman IL (2004). Haematopoietic stem cells adopt mature haematopoietic fates in ischaemic myocardium.. Nature.

[9] Toma C, Pittenger MF, Cahill KS, Byrne BJ, Kessler PD (2002). Human mesenchymal stem cells differentiate to a cardiomyocyte phenotype in the adult murine heart.. Circulation.

[10] Shake JG, Gruber PJ, Baumgartner WA, Senechal G, Meyers J (2002). Mesenchymal stem cell implantation in a swine myocardial infarct model: engraftment and functional effects.. Ann Thorac Surg.

[11] Muller-Ehmsen J, Peterson KL, Kedes L, Whittaker P, Dow JS (2002). Rebuilding a damaged heart: long-term survival of transplanted neonatal rat cardiomyocytes after myocardial infarction and effect on cardiac function.. Circulation.

[12] Reinecke H, Zhang M, Bartosek T, Murry CE (1999). Survival, integration, and differentiation of cardiomyocyte grafts: a study in normal and injured rat hearts.. Circulation.

[13] Huwer H, Winning J, Vollmar B, Welter C, Lohbach C (2003). Long-term cell survival and hemodynamic improvements after neonatal cardiomyocyte and satellite cell transplantation into healed myocardial cryoinfarcted lesions in rats.. Cell Transplant.

[14] Li RK, Jia ZQ, Weisel RD, Mickle DA, Zhang J (1996). Cardiomyocyte transplantation improves heart function.. Ann Thorac Surg.

[15] Soonpaa MH, Koh GY, Klug MG, Field LJ (1994). Formation of nascent intercalated disks between grafted fetal cardiomyocytes and host myocardium.. Science.

[16] Dengler J, Radisic M (2007). Tissue engineering approaches for the development of a contractile cardiac patch.. Future Cardiol.

[17] Dar A, Shachar M, Leor J, Cohen S (2002). Cardiac tissue engineering Optimization of cardiac cell seeding and distribution in 3D porous alginate scaffolds.. Biotechnology and bioengineering.

[18] Zimmermann WH, Melnychenko I, Wasmeier G, Didie M, Naito H (2006). Engineered heart tissue grafts improve systolic and diastolic function in infarcted rat hearts.. Nat Med.

[19] Yang L, Soonpaa MH, Adler ED, Roepke TK, Kattman SJ (2008). Human cardiovascular progenitor cells develop from a KDR+ embryonic-stem-cell-derived population.. Nature.

[20] Zhang J, Wilson GF, Soerens AG, Koonce CH, Yu J (2009). Functional cardiomyocytes derived from human induced pluripotent stem cells.. Circ Res.

[21] Langer R (2007). Editorial: tissue engineering: perspectives, challenges, and future directions.. Tissue Engineering.

[22] Radisic M, Park H, Martens TP, Salazar-Lazaro JE, Geng W (2007). Pre-treatment of synthetic elastomeric scaffolds by cardiac fibroblasts improves engineered heart tissue.. J Biomed Mater Res A.

[23] Naito H, Melnychenko I, Didie M, Schneiderbanger K, Schubert P (2006). Optimizing engineered heart tissue for therapeutic applications as surrogate heart muscle.. Circulation.

[24] Stevens KR, Kreutziger KL, Dupras SK, Korte FS, Regnier M (2009). Physiological function and transplantation of scaffold-free and vascularized human cardiac muscle tissue.. Proc Natl Acad Sci U S A.

[25] Dvir T, Kedem A, Ruvinov E, Levy O, Freeman I (2009). Prevascularization of cardiac patch on the omentum improves its therapeutic outcome.. Proc Natl Acad Sci U S A.

[26] Brown MA, Iyer RK, Radisic M (2008). Pulsatile perfusion bioreactor for cardiac tissue engineering.. Biotechnol Prog.

[27] Zandstra PW, Bauwens C, Yin T, Liu Q, Schiller H (2003). Scalable production of embryonic stem cell-derived cardiomyocytes.. Tissue Eng.

[28] Hattori F, Chen H, Yamashita H, Tohyama S, Satoh YS (2010). Nongenetic method for purifying stem cell-derived cardiomyocytes.. Nat Methods.

[29] Dubois NC, Craft AM, Sharma P, Elliott DA, Stanley EG (2011). SIRPA is a specific cell-surface marker for isolating cardiomyocytes derived from human pluripotent stem cells.. Nat Biotechnol.

[30] Perin EC, Dib N, Silva GV, DeMaria AN, Marroquin OC (2011). A Phase II Dose-Escalation Study of Allogeneic Mesenchymal Precursor Cells inPatients With Ischemic and Nonischemic Heart Failure.. American Heart Association.

[31] Iyer RK, Chui J, Radisic M (2009). Spatiotemporal tracking of cells in tissue-engineered cardiac organoids.. Journal of Tissue Engineering and Regenerative Medicine.

[32] AC N (1980). Study of non-muscle cells of the adult mammalian heart: a fine structural analysis and distribution.. Cytobios.

[33] Murthy SK, Sethu P, Vunjak-Novakovic G, Toner M, Radisic M (2006). Size-based microfluidic enrichment of neonatal rat cardiac cell populations.. Biomed Microdevices.

[34] Huang LR, Cox EC, Austin RH, Sturm JC (2004). Continuous Particle Separation through Deterministic Lateral Displacement.. Science.

[35] Inglis DW, Davis JA, Zieziulewicz TJ, Lawrence DA, Austin RH (2008). Determining blood cell size using microfluidic hydrodynamics.. J Immunol Methods.

[36] Green JV, Radisic M, Murthy SK (2009). Deterministic lateral displacement as a means to enrich large cells for tissue engineering.. Anal Chem.

[37] Inglis DW, Davis JA, Austin RH, Sturm JC (2006). Critical particle size for fractionation by deterministic lateral displacement.. Lab Chip.

[38] Cooper R, Lee L (2007). Chips & Tips: Preventing suspension settling during injection.. Lab on a Chip.

[39] Naito H, Melnychenko I, Didié M, Schneiderbanger K, Schubert P (2006). Optimizing Engineered Heart Tissue for Therapeutic Applications as Surrogate Heart Muscle.. Circulation.

[40] Heidi Au HT, Cui B, Chu ZE, Veres T, Radisic M (2009). Cell culture chips for simultaneous application of topographical and electrical cues enhance phenotype of cardiomyocytes.. Lab Chip.

[41] Dengler J, Song H, Thavandiran N, Masse S, Wood GA (2010). Engineered heart tissue enables study of residual undifferentiated embryonic stem cell activity in a cardiac environment.. Biotechnol Bioeng.

[42] McDonald JC, Duffy DC, Anderson JR, Chiu DT, Wu H (2000). Fabrication of microfluidic systems in poly(dimethylsiloxane).. Electrophoresis.

[43] Radisic M, Park H, Shing H, Consi T, Schoen FJ (2004). Functional assembly of engineered myocardium by electrical stimulation of cardiac myocytes cultured on scaffolds.. Proceedings of the National Academy of Sciences of the United States of America.

[44] Au HT, Cheng I, Chowdhury MF, Radisic M (2007). Interactive effects of surface topography and pulsatile electrical field stimulation on orientation and elongation of fibroblasts and cardiomyocytes.. Biomaterials.

[45] Tandon N, Cannizzaro C, Chao PH, Maidhof R, Marsano A (2009). Electrical stimulation systems for cardiac tissue engineering.. Nat Protoc.

