# Supplementary material for: Label-Free Enrichment of Functional Cardiomyocytes Using Microfluidic Deterministic Lateral Flow Displacement
Source: PLoS One. 2012 May 29;7(5):e37619. doi: 10.1371/journal.pone.0037619 (PMC3362623; doi:10.1371/journal.pone.0037619)
Supplement: Figure S1 — Analysis of cell output from syringes with and without magnetic stirring. (DOCX) [file pone.0037619.s001.docx]

Label-free enrichment of functional cardiomyocytes using microfluidic deterministic lateral flow displacement

Boyang Zhang, James Green, Shashi Murthy, Milica Radisic

**Supplements**

**Analysis of cell output from syringes with and without magnetic stirring.**

To analyze the cell output from the syringe during pumping with and without syringe mixing, both the output cell concentration and CMs percentage was determined as shown in Figure 1. The control represents the input cell mixture to the syringe. Cardiac cell mixture after one pre-plate was used here as the control. The initial cell solution was diluted to approximate 0.6 million cell/mL with CMs percentage of 70%.

P<0.01

Figure 1. Syringe output analysis.

Without syringe mixing, the amount of cell collected from the output was drastically lower, resulted in reduced cell concentration to 0.4 million cell/mL. In addition, since CMs settle fast due to their large size, they tend to get trapped within the syringe much more than the non-myocytes. This is indicated by the lower CMs percentage at the syringe output. However, by including mixing in the syringe all the cells were able to exit the syringe without settling. There were no significant differences in the output cell concentration and CMs percentage between the control and the condition with syringe mixing. This study indicates the importance of having a mixing mechanism in place to prevent cell settling during the experimental process.

**Statistical analysis.** Error bars in figures represent standard deviation. Statistical significance was determined using one-way ANOVA in conjunction with Tukey’s test. Normality and equality of variance were tested. p<0.05 were considered significant. A minimum of 3 samples were used per data point.
